# Genetic polymorphism and amino acid sequence variation in *Plasmodium falciparum* GLURP R2 repeat region in Assam, India, at an interval of five years

**DOI:** 10.1186/1475-2875-13-450

**Published:** 2014-11-21

**Authors:** Dinesh Kumar, Sunil Dhiman, Bipul Rabha, Diganta Goswami, Manab Deka, Lokendra Singh, Indra Baruah, Vijay Veer

**Affiliations:** Medical Entomology Division, Defence Research Laboratory, Tezpur, Assam 784001 India; Gauhati University, Guwahati, Assam India

**Keywords:** Malaria, *Plasmodium falciparum*, GLURP, Genetic diversity, Amino acid, Repeated sequence

## Abstract

**Background:**

The R2 repeat region of GLURP has been reported as a good genetic marker for *Plasmodium falciparum* genotyping. Proper knowledge of the extent and nature of *P. falciparum* genetic diversity using highly immunogenic R2 repeat region in malaria-endemic areas is a crucial element to understand various aspects related to immunity acquisition and disease pathogenesis.

**Methods:**

Population diversity of *P. falciparum* GLURP and amino acid sequence repeats in GLURP R2 region was studied in malaria-endemic Assam state, northeast India and compared at an interval of five years during 2005 (Group-A) and 2011 (Group-B).

**Results:**

Of the 66 samples, a total of 55 samples showed positive PCR bands for GLURP R2 region and altogether ten types of alleles with size ranging from 501 bp to 1,050 bp (50 bp bin) were observed and coded as genotypes I-X. In Group-A (n = 29), 24 samples were found infected with single, four with double and one with triple *P. falciparum* genotype, while in Group-B (n = 26), single genotype was found in 23 samples, double in two samples and triple in one sample. Genotype IV showed significant increase (p = 0.002) during 2011 (Group-B). Genotypes I to V were more common in Group-B (62%), however genotypes VI to X were more frequently distributed in Group-A. The expected heterozygosity was found slightly higher in Group-A (H_E_ = 0.87) than Group-B (H_E_ = 0.85), whereas multiplicity of infection (MOI) in Group-A (MOI = 1.21 ± 0.49) and Group-B (MOI = 1.12 ± 0.43) did not display significant variation. The amino acid repeat sequence unit (AAU) DKNEKGQHEIVEVEEILPE (called ‘a’) was more frequent in the well-conserved part of R2 repeat region.

**Conclusion:**

The present study is the first extensive study in India which has generated substantial data for understanding the type and distribution of naturally evolved genetic polymorphism at amino acid sequence level in GLURP R2 repeat region in *P. falciparum*. There was decrease in the PCR amplicon size as well as the number of AAU [amino acid repeat unit] in Group-B displaying the bottleneck effect. The present study described a new type of AAU ‘d’ which varied from the other previous known AAUs.

**Electronic supplementary material:**

The online version of this article (doi:10.1186/1475-2875-13-450) contains supplementary material, which is available to authorized users.

## Background

GLURP (glutamate rich protein) is a 220-KDa exo-antigen expressed in both pre-erythrocytic and erythrocytic stages of *Plasmodium falciparum*, as well as on the surface of newly released merozoites in human host [1]. It is highly immunogenic and serves as target for antibodies involved in antibody-dependent cellular inhibition (ADCI) in the presence of monocytes [2]. In ADCI, the cytophilic antibodies (mainly IgG3 and IgG1) act in synchronization with monocytes to control *Plasmodium* multiplication [3]. Immuno-epidemiological studies conducted in different endemic areas have showed high levels of GLURP antibodies in malaria-infected humans, which were found to provide protection against high parasitaemia and clinical disease [4, 5]. The gene encoding GLURP in *P. falciparum* consists of three regions namely, N-terminal non-repetitive region (R0), central repetitive region (R1) and an immunodominant C-terminal repetitive region (R2) [1]. The recombinant *P. falciparum* GLURP non-repeat R0 and repeat R2 regions expressed in *Escherichia coli* were found to induce a humoral response in vivo [6]. A recent study on naive Dutch volunteers have shown that antibody level against R2 repeat region of GLURP in plasma was found much higher than that of R1 and R0 regions [7]. The studies further suggested that R2 repeat region plays an important role in inducing protective immunity against *P. falciparum* malaria. The R2 repeat region has been reported as a good genetic marker for *P. falciparum* genotyping and also for differentiating new infection from recurring infections [8]. Therefore, knowledge of the extent and nature of *P. falciparum* genetic diversity in malaria-endemic areas is a crucial element in understanding various aspects related to immunity acquisition and disease pathogenesis.

Northeastern states of India report high *P. falciparum* cases and malaria-attributed deaths annually [9, 10]. The entire northeast region has unique geography conducive to malaria spread and also its poor socio-economic conditions increase transmission risk [11–13]. In India, *P. falciparum* population diversity has been studied using MSP1, MSP2 and CSP markers [14–16], however, no study on diversity of GLURP R2 has been reported. The current study was carried out in the malaria-endemic Assam state of northeast India to understand the population diversity of *P. falciparum* GLURP and amino acid sequence repeats in GLURP R2 region using the samples collected during the peak malaria periods in 2005 and 2011.

## Methods

### Study site

The present study was conducted in the forest-fringed Orang public health centre (PHC) area (92°10’40”E longitude to 92°21’20”N latitude) of Udalguri district in Assam. The study area has many tea gardens and evergreen dense forests and experiences high annual rainfall (1,500-2,000 mm), and humidity (82-88%). The temperature ranges from 13.5°C in winter to 34.5°C in summer. The area reports perennial malaria and the inhabitants are socio-economically backward mixed population of Bodos, Nepalese, Adivasis and Rabhas [12, 13]. The blood samples for the present study were collected during April-September in 2005 and 2011.

### Sample collection and DNA extraction

Malaria-positive samples were collected from PHC and health camps organized by Assam State Government health department. Patients with clinical symptoms of malaria were examined using malaria rapid detection kit (Optimal IT^®^, Diamed GmbH, Switzerland and First Sign^®^ Avantor Performance Materials India, Pvt Ltd) in the field while smears were obtained on glass slide for microscopy. A few drops of blood from rapid detection kit confirmed *P. falciparum* infections were taken in FTA™ classic cards (Whatman, Sweden) for molecular analysis. The blood-blotted FTA™ cards were air dried, labelled and stored in dried condition. For long-term storage, FTA™ cards were stored in airtight containers in -20°C. Microscopy confirmed dried FTA™ card samples were used to extract DNA for PCR diagnosis of malaria parasites. A small portion of the blood-blotted FTA cards were cut using Harris Micro Punch™ (Whatman, USA) and taken in a 2.0 ml centrifuge tube. DNA extraction was carried out using Qiagen Blood Mini Kit (Qiagen AG, Germany) following the standard manufacturer protocol. In the present study, 38 samples were collected in 2005 (Group-A) while 46 sample were collected in 2011 (Group-B). Mean age (mean ± SD) of the study participants for Group-A was 22 ± 2.9 years and for Group-B was 13 ± 1.6 years. The male/female ratio in Group-A and Group-B samples was 1.06 and 1.36, respectively.

### PCR assay

All samples were put for species-specific nested PCR assay for detection of the different *Plasmodium* species, following the reaction conditions described previously [9, 17]. The samples confirmed for *P. falciparum* were genotyped for polymorphic GLURP R2 repeat region by semi-nested PCR reaction using the following set of primers as described elsewhere [18].

GOF: 5′TGAATTTGAAGATGTTCACACTGAAC3′;

GOR: 5′ GTGGAATTGCTTTTTCTTCAACACTAA3′ and

GNF: 5′TGTTCACACTGAACAATTAGATTTAGATCA 3′

The resultant amplicons were electrophoresed in 2% agarose gel (Sigma Aldrich, USA) and PCR product sizes were determined using 50 bp DNA marker (Sigma Aldrich, USA) under UV transilluminator system (Syngene G-Box, UK). DNA fragments obtained were grouped into 50 bp bins and each band of distinct size was considered as different allele. Selected amplicons from each year were purified using GenElute™ gel extraction kit (Sigma Aldrich, USA) according to the manufacturer’s protocol. Purified products were then sequenced at Biolink Pvt Ltd, New Delhi, India using an ABI3730XL DNA sequencer (Applied Biosystem) with GNF and GOR primers, respectively.

### Sequence analysis

Alignment of nucleotide sequences and translation to reverse complementary sequences were carried out using BioEdit computer program (v7.1.3.0). Comparison of aligned nucleotide sequences was made with those available in NCBI’s GenBank database using BLASTn with program selection optimized for highly similar sequences (megablast). The newly generated sequences were deposited in GenBank (accession number: KJ664815-29, KJ664831-36, KJ645966 and KJ719442). Nucleotide sequences were translated using Expasy online software and the obtained amino acid sequences were compared with available sequences in the NCBI database (accession number: XM001347592, M59706, AF247634, AF191065-67, AY138510-11) using CLUSTAL-W of the MEGA 5.1 software package [19] and manually adjusted wherever necessary.

### Statistical analysis

The allelic frequency was calculated by dividing the number of a particular allele by the total number of samples positive for that allelic family of the gene [20]. The multiplicity of infection (MOI) was calculated by dividing the total number of fragments detected in GLURP by the number of samples positive for the same marker. Heterozygosity, which represents the probability of being infected by two parasites with different alleles at a given locus and ranging between 0 and 1, was calculated by using the following formula- H_E_ = [n/(n - 1)][(1 - ∑pi^2^)], where ‘n’ is the number of isolates sampled and ‘pi’ is the allele frequency at a given locus [21]. Isolates with more than one genotype were considered as polyclonal infection, while the presence of a single allele was considered as monoclonal infection. Quantitative data were expressed as the mean ± standard deviation (SD) and were compared using Student’s unpaired ‘t’ test. Qualitative data were compared using Fisher’s exact test. Statistical analysis was performed using GraphPad InStat (Ver. 3.05, USA).

### Ethical consideration

The study protocol was approved by the Medical Ethics Committee of LGB Regional Institute of Mental Health (under Government of India, Ministry of Health and Family Welfare) Tezpur, Assam, India. Participants were clearly explained the objectives of the study and enrolled for the study only after consent.

## Results

### Allelic diversity

**Figure 1:**
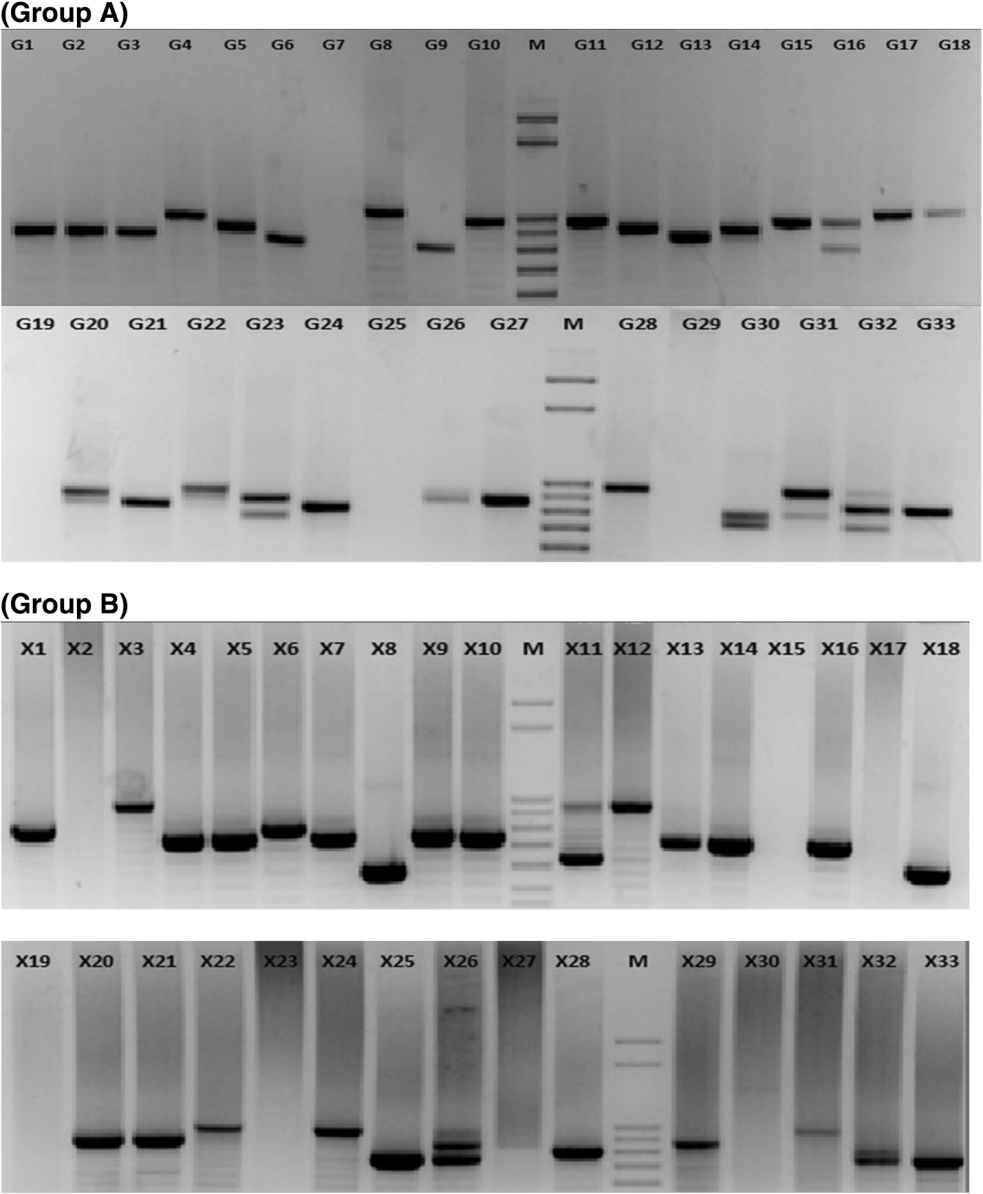
**Molecular size variation of GLURP R2 repeat regions of** ***Plasmodium falciparum*** **field isolates (Group A and B).**

**Figure 2:**
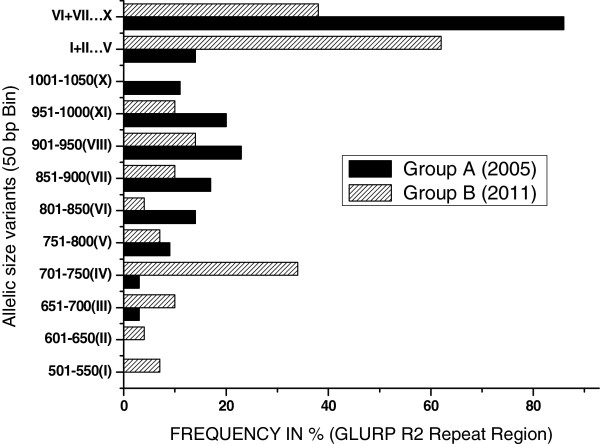
**Frequency (%) distribution of allelic size variants of** ***P. falciparum*** **GLURP R2 repeat region in Group-A (collected in 2005) and Group-B (collected in 2011).**

Altogether, 84 samples were analysed in the present study, of which 38 were collected in 2005 (Group-A) and 46 in 2011 (Group-B). The age (mean ± SD) of the participants of Group-A was 22 ± 2.9 years while Group-B was 13 ± 1.6 years. Out of 84 samples, 66 confirmed for *P. falciparum* mono-infection were used for GLURP genotyping, of which 55 samples showed positive PCR amplification for GLURP R2 region. Ten types of different alleles with size ranging from 501 bp to 1,050 bp (50 bp bin) were observed and coded as genotypes I-X (Table 1). Of the 66 samples, 29 from Group-A and 26 from Group-B were successfully genotyped for GLURP R2 locus (Figure 1). In Group-A (n = 29), 24 samples were found infected with single, four with double and one with triple *P. falciparum* genotype (Figure 1a). In Group-B (n = 26), a single genotype was found in 23 samples, double in two samples and triple in one sample (Figure 1b). Overall, eight types of genotype in Group-A and nine in Group-B were found in the present study. In Group-A, genotype I and II were not found while in Group-B genotype X could not be recorded. Frequency of genotype IV was high in Group-A (34%), while genotype VIII was most frequently (23%) found among the samples in Group-B. Genotype IV showed significant increase (p = 0.002) during 2011 (Group-B) whereas the other genotypes did not show significant variation between the two study years (p > 0.05) (Figure 2 and Table 1). Further, the genotypes I to V (those size <801 bp) were found to be more common in Group-B (62%), while the genotypes VI to X (size ≥801 bp) were more frequently distributed in Group-A (86%). The expected heterozygosity was found slightly higher in Group-A (H_E_ = 0.87) compared to Group-B (H_E_ = 0.85). Similarly, the MOI in Group-A (MOI = 1.21 ± 0.49) and Group-B (MOI = 1.12 ± 0.43) did not display significant variation.

**Table 1:** **Allelic size variants of GLURP R II repeat region of** ***P. falciparum*** **field isolates in Group-A (collected in 2005) and Group-B (collected in 2011)**

Types	Allelic size variants (50 bp bin)	Group-A (N = 29) n (%)	Group-B (N = 26) n (%)	Total (N = 55) N’ (%)	p value
I	501-550	0	2(7)	2(3)	0.2014
II	601-650	0	1(4)	1(2)	0.4531
III	651-700	1(3)	3(10)	4(6)	0.3211
IV	701-750	1(3)	10(34)	11(17)	0.0016
V	751-800	3(9)	2(7)	5(8)	1
VI	801-850	5(14)	1(4)	6(9)	0.209
VII	851-900	6(17)	3(10)	9(14)	0.4943
VIII	901-950	8(23)	4(14)	12(19)	0.5221
IX	951-1000	7(20	3(10)	10(16)	0.3268
X	1001-1050	4(11)	0	4(6)	0.1198
	I + II + III + IV + V	5(14)	18(62)	23(36)	0.0001
	VI + VII + VIII + IX + X	30(86)	11(38)	41(64)	0.0001
Total		35(100)	29(100)	64(100)	0.3768
MOI		1.21 ± 0.49	1.12 ± 0.43	1.16 ± 0.46	

N = number of sample; n = frequency of GLURP allelic size variants;

% = percentage of GLURP allelic size variants; MOI = multiplicity of infection.

### Amino acid sequence diversity of GLURP R2 repeat region

R2 locus of GLURP gene from randomly selected samples from both the groups (Group-A = 9; Group-B = 14) were sequenced. There was considerable variation in the length of R2 repeat region among the isolates of both groups (Figure 1a and b). The amino acid repeat sequence unit (AAU) DKNEKGQHEIVEVEEILPE (called ‘a’) was present more frequently in the well-conserved part of R2 repeat region. Apart from this, three other types of AAU were also detected in which GQ [glycine-glutamine] amino acid positions at sixth and seventh were replaced by amino acids VE [valine-glutamic acid] (called ‘b’), VQ [valine-glutamine] (called ‘c’), and additionally a new type of AAU was found, where amino acids VE were present in sixth and seventh position, while amino acid D [aspartic acid] was found in 14^th^ position (called ‘d’). However, an extra D or E amino acid was found in between some AAUs (Additional file 1). While comparing with the available NCBI database sequences, a total 20 different types of haplotypes were observed and designated as T1-T20 (Additional file 2). Six and eight different haplotypes were observed in Group-A and B, respectively, while NCBI database showed seven haplotypes only. Haplotype T8 was common in both the groups and found in maximum number of samples (n = 3) as compared to T9 (n = 2), T10 (n = 1), T11 (n = 1), T12 (n = 1) and T13 (n = 1) in Group-A. However in Group-B T14 (n = 4) and T16 (n = 4) haplotypes were found in high number (Additional file 2).

The arrangement of repeat amino acid unit varied among the isolates, however in the first four position, b-a-c-a arrangement was found in majority of the samples in Group-A (n = 7; 77.8%) compared to Group-B (n = 7; 50%) (Additional file 1). However in NCBI [National Centre for Biotechnology Information] sequences only one sequence had b-a-c-a arrangement of amino acid unit. In all the three groups (reference NCBI sequences, Group-A and B) b-a-c-a and b-c-c-a arrangements were common, whereas b-c-a-c arrangement was unique in Group-A, b-a-d-a and b-a-a-a were unique in Group-B, and b-c-c-c, b-c-b-a and .-.-c-a repeat amino acid unit arrangements were unique in the NCBI sequences. All the samples taken currently and NCBI sequences contained ‘a’ type AAU in high frequency as compared to b, c and d type AAUs. The ‘d’ type of AAU was present in Group-B only and could not be found in Group-A and NCBI sequences.

In the present study, a total of seven types of amino acid repeat pair order (AAUO) were found and designated as ‘T’ to ‘Z’, with an extra presence of 20^th^ amino acid either D (aspartate) or E (glutamate) located in between the repeat units as shown in Additional file 2. Of the seven AAUOs, six types were common in both the Groups and NCBI sequences, except ‘T’ type which was found in Group-B only. The ‘Y’ type AAUO was found in high number in all the groups as compared to other AAUO (T, U, V, W, X, and Z) (Additional file 2). However the average of ‘Y’ per sample in Group-A (2.6 ± 1.4) was statistically high than Group-B (1.2 ± 1.0) (p = 0.01), while ‘V’ was found to be higher in Group-B (0.5 ± 0.5) than Group-A (0.1 ± 0.3) (p = 0.04). Among the extra ‘D’ and ‘E’ amino acids, only ‘E’ showed significant variation between Group-A and -B (p = 0.03). Further, while comparing total AAUs, AAUOs and presence of extra ‘D’ and ‘E’ amino acids in isolates of Group-A and B, the mean AAUOs were significantly higher in Group-A (4.2 ± 0.8) than Group-B (3.4 ± 0.8) (p = 0.03). The comparison of amino acid repeat sequences of total samples from both the experiment Groups (A and B), with the reference NCBI sequences, revealed that the average frequency distribution of AAUO ‘U’ was stastically higher in the reference sequences (0.3 ± 0.5) (p = 0.04) (Additional file 2).

## Discussion

The current study has suggested considerable polymorphism and variation in the genetic sequences of *P. falciparum* GLURP R2 repeat region among the samples collected after an interval of five years in a *P. falciparum* malaria-endemic setting in the northeastern region of India. Many studies have indicated that genetic diversity and multiplicity of *Plasmodium* species was responsible for the biological success of the malaria parasite [22–25]. Genetic polymorphism not only confers the malaria parasite with the ability to evade human immune response but also helps in developing resistance to anti-malarial drugs [24, 25]. *Plasmodium falciparum* has shown decreased susceptibility to all major anti-malarial drugs in different regions of the world [10, 26, 27]. Various malaria vaccine candidate antigens are in different stages of clinical trials, of which circumsporozoite protein (CSP) antigen-based RTS,S vaccine is under phase-3 trial and the results have showed moderate efficacy against both clinical and severe malaria among the young infants [28]. However, significant heterogeneity in vaccine efficacy was seen across different trial sites [29], probably due to genetic polymorphism of *P. falciparum* CSP gene in different geographical regions [30].

Malaria parasite *P. falciparum* has many polymorphic loci that have been used to study genetic diversity [21, 22, 30]. GLURP has been considered an important antigen that has been playing an important role in the induction of protective immunity against *P. falciparum* malaria [7]. However, only a few studies have been conducted on conserved amino acid sequences of R2 repeat region of GLURP from field sample isolates of *P. falciparum,* but none of the studies has reported the polymorphism and amino acid repeat order of GLURP in India [31]. The present study is first extensive attempt to understand the: (i) overall population diversity of *P. falciparum*; (ii) changes in the population within this time period; and, (iii) arrangement of amino acid sequence pair repeat in R2 region of *Pf* GLURP gene over the time interval of five years in the study area. There has been a non-significant decrease in the MOI in 2011, but a genetic drift in *P. falciparum* population over the time, which could be due to the change in the anti-malarial drug policy in northeast India during 2007–08 [32, 33].

Previous studies have reported two GLURP (R2) alleles in Honduras [34], three in French Guyana [35] and each four in Colombia [36] and Brazil [37] in the area of low endemicity; however in high endemicity areas in Africa and Asia, anywhere from eight in India to twenty GLURP alleles in Sudan were reported respectively [18, 20, 21, 38]. Current study found eight alleles in Group-A and nine alleles in Group-B, whereas overall ten alleles were found in the total samples compared to eight reported in Orissa [20]. The present study has emphasized that the amplicons size of R2 repeat region in Group-A was larger than Group-B, but the frequency of genotype IV was found to increase in Group-B. A previous study suggested that increase in allele frequency may arise as a result of selection induced due to drug pressure and other control strategies [39].

The results suggest that amino acid sequence DKNEKGQHEIVEVEEILPE ‘a’ was present more frequently in the well-conserved part of *P. falciparum* GLURP R2 repeat region. Present results were similar to a previous study, which showed that GQ in sixth and seventh position is replaced by VE ‘b’ and VQ ‘c’ , respectively [1]. However, the present study has found a new type of amino acid sequence pair VE in sixth and seventh position and D in 14^th^ position and termed as ‘d’. There were thirteen different haplotypes found among the twenty three sequenced isolates when compared with the amino acid sequence of 3D7 as standard sequence in the present study [40]. On comparing present sequences with the previously published sequences [1, 40], a maximum number of conserved repeat amino acid pairs were found in Group-A (n = 12) compared to Group-B (n = 11) and NCBI sequences (n = 11). Many studies have reported that the conserved repeat sequences were found to vary among the isolates [20, 30, 37]. There were thirteen different types of haplotype found in the entire field samples, while only seven haplotypes were found in NCBI sequences, of which about 50% contained b-a-c-a in the first four positions. The haplotypes found in NCBI sequences (T1-T7) were different from each other as well as from the present sequences. The most common AAU found in the two Groups was ‘a’ (DKNEK*GQ*HEIVEV*E*EILPE) type followed by ‘c’ (DKNEK*VQ*HEIVEV*E*EILPE), ‘b’ (DKNEK*VE*HEIVEV*E*EILPE) and d’ (DKNEK*VE*HEIVEV*D*EILPE), respectively. Similar to the present results, the ‘a’ type AAU was also predominant among the NCBI sequences. On the other hand, ‘d’ was present in four samples in Group-B but not found in NCBI sequences. The most predominant type of AAUO found in present samples as well as in the NCBI sequences was ‘Y’ (c-a) type, however ‘T’ (d-a) type was present in four samples in Group-B only. The results indicate that there has been increase in the frequency of sample having ‘d’ AAU and ‘T’ AAUO during 2011, however many of the AAU and AAUO did not show variation, suggesting that the sequence units and orders are homologues with the NCBI sequences from other countries. A previous study conducted on sequence analysis of GLURP in China has revealed that the native sequences were similar to the overseas sequences, which suggests the sequences homology [31]. The conservation in sequences could efficiently be used to target *P. falciparum* in different malaria-endemic areas globally. The present study however revealed that the number of AAU did not differ over the period of time but the arrangement of AAUO differed, which can be attributed to the change in the anti-malarial drug policy for northeastern states during 2007–08. In the previous studies similar observations were recorded in Malawi where the chloroquine wild genotype was restored after thirteen years of withdrawal of chloroquine from malaria control programmes [41].

The present study is the first extensive study in India which has generated substantial data for understanding the type and distribution of naturally evolved genetic polymorphism at amino acid sequence level in GLURP R2 repeat region. The PCR amplicons size as well as the number of AAU decreases in the samples of Group-B, displaying the bottleneck effect under anti-malarial drug pressure. Sequence analysis of selected samples showed considerable haplotypic diversity. The present study described a new type of AAU ‘d’ which varied from other previously known AAUs (a, b and c). The amino acid repeat pattern at first four positions was common in both the experiment Groups and NCBI sequences. Present data could also be useful in monitoring the *P. falciparum* population in order to check new strains. Amino acid repeat pattern of GLURP R2 repeat region of *P. falciparum* from other countries would be useful in understanding diversity and identifying homology among the different isolates.

## Electronic supplementary material

Additional file 1:**Distribution of amino acid repeat unit (AAU) of GLURP R2 region of field isolates and NCBI sequences of** ***Plasmodium.***(DOCX 35 KB)

Additional file 2:**Arrangement of amino acid repeat order (AAUO) and haplotype diversity of GLURP R 2 region of** ***P. falciparum*** **in field isolates and NCBI sequence database.**(DOCX 39 KB)

## References

[1] Borre MB, Dziegiel M, Hogh B, Petersen E, Rieneck K, Riley E, Meis JF, Aikawa M, Nakamura K, Harada M, Wind A, Jakobsen PH, Cowland J, Jepsen S, Axelsen NH, Vuust J (1991). Primary structure and localization of a conserved immunogenic *Plasmodium falciparum* glutamate rich protein (GLURP) expressed in both the preerythrocytic and erythrocytic stages of the vertebrate life cycle. Mol Biochem Parasitol.

[2] Oeuvray C, Theisen M, Rogier C, Trape JF, Jepsen S, Druilhe P (2000). Cytophilic immunoglobulin responses to *Plasmodium falciparum* glutamate-rich protein are correlated with protection against clinical malaria in Dielmo, Senegal. Infect Immun.

[3] Tebo AE, Kremsner PG, Luty AJ (2001). *Plasmodium falciparum*: a major role for IgG3 in antibody-dependent monocyte-medeated cellular inhibition of parasite growth in vitro. Exp Parasitol.

[4] Lusingu JP, Vestergaard LS, Alifrangis M, Mmbando BP, Theisen M, Kitua AY, Lemnge MM, Theander TG (2005). Cytophilic antibodies to *Plasmodium falciparum* glutamate rich protein are associated with malaria protection in an area of holoendemic transmission. Malar J.

[5] Nebie I, Diarra A, Ouedraogo A, Soulama I, Bougouma EC, Tiono AB, Konate TA, Chilengi R, Theisen M, Dodoo D, Remarque E, Bosomprah S, Milligan P, Sirima SB (2008). Humoral responses to *Plasmodium falciparum* blood-stage antigens and association with incidence of clinical malaria in children living in an area of seasonal malaria transmission in Burkina Faso, West Africa. Infect Immun.

[6] Theisen M, Vuust J, Gottschau A, Jepsen S, Hogh B (1995). Antigenicity and immunogenicity of recombinant glutamate-rich protein of *Plasmodium falciparum* expressed in *Escherichia coli*. Clin Diagn Lab Immunol.

[7] Turner L, Wang CW, Lavstsen T, Mwakalinga SB, Sauerwein RW, Hermsen CC, Theander TG (2011). Antibodies against PfEMP1, RIFIN, MSP3 and GLURP are acquired during controlled *Plasmodium falciparum* malaria infection in naive volunteers. PLoS One.

[8] Barrera SM, Perez MA, Knudson A, Nicholls RS, War AP (2010). Genotyping of *Plasmodium falciparum* by multiplex PCR using gene msp1, msp2 and glurp, in four locations in Colombia. Biomedica.

[9] Dhiman S, Goswami D, Kumar D, Rabha B, Sharma DK, Bhola RK, Baruah I, Singh L (2013). Nested PCR detection of *Plasmodium malariae* from microscopically confirmed *Plasmodium falciparum* samples in endemic area of Northeast India. Folia Parasitol.

[10] Goswami D, Dhiman S, Rabha B, Kumar D, Baruah I, Sharma DK, Veer V (2014). *Pfcrt* mutant haplotypes may not correspond to the chloroquine resistance. J Infect Dev Ctries.

[11] Nath MJ, Bora AK, Yadav K, Talukdar PK, Dhiman S, Baruah I, Singh L (2013). Prioritizing areas for malaria control using geographical information system in an endemic district of Assam, India. Pub Health.

[12] Yadav K, Dhiman S, Rabha B, Veer V (2014). Socio-economic determinants for malaria transmission risk in an endemic primary health centre in Assam, India. Inf Dis Pov.

[13] Yadav K, Nath MJ, Talukdar PK, Saikia PK, Baruah I, Singh L (2011). Malaria risk areas of Udalguri district of Assam, India: a GIS-based study. Int J Geogr Inf Sci.

[14] Singh JP, Verma S, Bhattacharya PR, Srivastava N, Dash AP, Biswas S (2009). *Plasmodium falciparum* circumsporozoite protein: epidemiological variations among field isolates prevalent in India. Trop Med Int Health.

[15] Baruah S, Lourembam SD, Sawian CE, Baruah I, Goswami D (2009). Temporal and spatial variations in MSP1 clonal composition of *Plasmodium falciparum* in districts of Assam, Northeast India. Infect Genet Evol.

[16] Joshi H, Valecha N, Verma A, Kaul A, Mallick PK, Shalini S, Prajapati SK, Sharma SK, Dev V, Biswas S, Nanda N, Malhotra MS, Subbarao SK, Dash AP (2007). Genetic structure of *Plasmodium falciparum* field isolates in eastern and north-eastern India. Malar J.

[17] Dhiman S, Bhola RK, Goswami D, Rabha B, Kumar D, Baruah I, Singh L (2012). Polymerase chain reaction detection of human host preference and *Plasmodium* parasite infections in field collected potential malaria vectors. Pathog Global Health.

[18] Snounou G, Zhu X, Siripoon N, Jarra W, Thaithong S, Brown NK, Viriyakosol S (1999). Biased distribution of MSP 1 and MSP 2 allelic variants in *Plasmodium falciparum* population in Thailand. Trans R Soc Trop Med Hyg.

[19] Tamura K, Peterson D, Peterson N, Stecher G, Nei M, Kumar S (2011). MEGA5: molecular evolutionary genetics analysis using maximum likelihood, evolutionary distance, and maximum parsimony methods. Mol Biol Evol.

[20] Ranjit MR, Das A, Das BP, Das BN, Dash BP, Chhotray GP (2005). Distribution of *Plasmodium falciparum* genotypes in clinically mild and severe malaria cases in Orissa, India. Trans R Soc Trop Med Hyg.

[21] Mwingira F, Nkwengulila G, Schoepflin S, Sumari D, Beck HP, Snounou G, Felger I, Olliaro P, Mugittu K (2011). *Plasmodium falciparum msp1, msp2* and *glurp* allele frequency and diversity in sub-Saharan Africa. Malar J.

[22] Ranjit MR, Sharma YD (1999). Genetic polymorphism of *falciparum* malaria vaccine candidate antigen genes among field isolates in India. Am J Trop Med Hyg.

[23] Ekland EH, Fidock DA (2007). Advances in understanding the genetic basis of antimalarial drug resistance. Curr Opin Microbiol.

[24] Casares S, Richie LT (2009). Immune evasion by malaria parasites: a challenge for vaccine development. Curr Opin Immunol.

[25] Castellini MA, Buguliskis JS, Casta LJ, Butz CE, Clark AB, Kunkel TA, Taraschi TF (2011). Malaria drug resistance is associated with defective DNA mismatch repair. Mol Biochem Parasitol.

[26] Wongsrichanalai C, Pickard AL, Wernsdorfer WH, Meshnick SR (2002). Epidemiology of drug-resistant malaria. Lancet Inf Dis.

[27] Dondorp AM, Nosten F, Yi P, Das D, Phyo AP, Tarning J, Lwin KM, Ariey F, Hanpithakpong W, Lee SJ, Ringwald P, Silamut K, Imwong M, Chotivanich K, Lim P, Herdman T, An SS, Yeung S, Singhasivanon P, Day NP, Lindegardh N, Socheat D, White NJ (2009). Artemisinin resistance in *Plasmodium falciparum* malaria. N Engl J Med.

[28] Agnandji ST, Lell B, Fernandes JF, Abossolo BP, Methogo BG, Kabwende AL, Adegnika AA, Mordmüller B, Issifou S, Kremsner PG, Sacarlal J, Aide P, Lanaspa M, Aponte JJ, Machevo S, Acacio S, Bulo H, Sigauque B, Macete E, Alonso P, Abdulla S, Salim N, Minja R, Mpina M, Ahmed S, Ali AM, Mtoro AT, Hamad AS, Mutani P, Tanner M (2012). A Phase 3 Trial of RTS, S/AS01 malaria vaccine in African infants. N Engl J Med.

[29] Agnandji ST, Lell B, Fernandes JF, Abossolo BP, Kabwende AL, Adegnika AA, Mordmüller B, Issifou S, Kremsner PG, Loembe MM, Sacarlal J, Aide P, Madrid L, Lanaspa M, Mandjate S, Aponte JJ, Bulo H, Nhama A, Macete E, Alonso P, Abdulla S, Salim N, Mtoro AT, Mutani P, Tanner M, Mavere C, Mwangoka G, Lweno O, Juma OA, Shekalaghe S (2014). Efficacy and safety of the RTS, S/AS01 malaria vaccine during 18 months after vaccination: a phase 3 randomized, controlled trial in children and young infants at 11 African sites. RTS, S clinical trials partenership. PLoS Med.

[30] Zeeshan M, Alam MT, Sumiti V, Bora H, Tyagi RK, Alam MS, Choudhary V, Mittra P, Lumb V, Bharti PK, Udhayakumar V, Singh N, Jain V, Singh PP, Sharma YD (2012). Genetic variation in the *Plasmodium falciparum* circumsporozoite protein in India and its relevance to RTS S malaria vaccine. PLoS One.

[31] Ping-Xin Z, Mei-Xin Z, Lei Z, Ping-Ya Y, Xin G (2002). Sequence analysis and genotypes of glutamate rich protein of *Plasmodium falciparum* isolates from different malaria endemic areas in China. Biomed Environ Sci.

[32] Katoch VM, Satyanarayana K, Srivastava VK, Kant L, Saha B (2009). Integrated disease vector control of malaria. A success story based in Assam, Northeastern India. ICMR Bull.

[33] Anvikar AR, Arora U, Sonal GS, Mishra N, Shahi B, Savargaonkar D, Kumar N, Shah NK, Valecha N (2014). Antimalarial drug policy in India: past, present & future. Indian J Med Res.

[34] Haddad D, Snounou G, Mattei D, Enamorado IG, Figueroa J, Stahl S, Berzins K (1999). Limited genetic diversity of *Plasmodium falciparum* in field isolates from Honduras. Am J Trop Med Hyg.

[35] Ariey F, Chalvet W, Hommel D, Peneau C, Hulin A, Mercereau-Puijalon O, Duchemin JB, Sarthou JL, Reynes JM, Fandeur T (1999). *Plasmodium falciparum* parasites in French Guiana: limited genetic diversity and high selfing rate. Am J Trop Med Hyg.

[36] Montoya L, Maestre A, Carmona J, Lopes D, do Rosario V, Blair S (2003). *Plasmodium falciparum*: diversity studies of isolates from two Colombian regions with different endemicity. Exp Parasitol.

[37] Pratt- Riccio LR, Perce-da-Silva Dde S, Lima- Junior JC, Theisen M, Santos F, Daniel-Ribeiro CT, de Oliveira-Ferreira J, Banic DM (2013). Genetic polymorphism in glutamate-rich protein of *Plasmodium falciparum* field isolates from a malaria-endemic area of Brazil. Mem Inst Oswaldo Cruz.

[38] A-Elbasit IE, A-Elgadir TM, Elghazali G, Elbashir MI, Giha HA (2007). Genetic fingerprints of parasites causing severe malaria in a setting of low transmission in Sudan. J Mol Microbiol Biotechnol.

[39] Jongwutiwesa S, Putaporntipa C, Hughesb AL (2010). Bottleneck effects on vaccine-candidate antigen diversity of malaria parasites in Thailand. Vaccine.

[40] Gardner MJ, Hall N, Fung E, White O, Berriman M, Hyman RW, Carlton JM, Pain A, Nelson KE, Bowman S, Paulsen IT, James K, Eisen JA, Rutherford K, Salzberg SL, Craig A, Kyes S, Chan MS, Nene V, Shallom SJ, Suh B, Peterson J, Angiuoli S, Pertea M, Allen J, Selengut J, Haft D, Mather MW, Vaidya AB, Martin DM (2002). Genome sequence of the human malaria parasite *Plasmodium falciparum*. Nature.

[41] Laufer MK, Thesing PC, Eddington ND, Masonga R, Dzinjalamala FK, Takala SL, Taylor TE, Plowe CV (2006). Return of chloroquine antimalarial efficacy in Malawi. N Engl J Med.

