# Supplementary material for: Genetic polymorphism and amino acid sequence variation in Plasmodium falciparum GLURP R2 repeat region in Assam, India, at an interval of five years
Source: Malar J. 2014 Nov 21;13:450. doi: 10.1186/1475-2875-13-450 (PMC4256832; doi:10.1186/1475-2875-13-450)
Supplement: Supplementary file 1 — Additional file 1:Distribution of amino acid repeat unit (AAU) of GLURP R2 region of field isolates and NCBI sequences of Plasmodium.(DOCX 35 KB) [file 12936_2014_3613_MOESM1_ESM.docx]

|  | **Amino acid sequence diversity in R 2 repeat region of *P. falciparum* GLURP** | | | | | | | | | | | | | | | | | | | | | | | | | | |  | | |  |
| --- | --- | --- | --- | --- | --- | --- | --- | --- | --- | --- | --- | --- | --- | --- | --- | --- | --- | --- | --- | --- | --- | --- | --- | --- | --- | --- | --- | --- | --- | --- | --- |
|  |  | **851** | **Repeat Region** | | | | | | | | | | | | | | | | | | | | | | | | | **1065** | | |  |
| **NCBI sequences** | **3D7** | **N N E F Q E I N E D** | **1** | **D** | **b** | **_** | **c** | **_** | **b** | **_** | **a** | **_** | **c** | **_** | **a** | **_** | **b** | **_** | **a** | **D** | **a** | **.** | **.** | **.** | **.** | **.** | **.** | | **I V E I E E V P S Q T N N** | | |
|  | **AF247634** | **N N E F Q E I N E D** | **1** | **D** | **b** | **_** | **c** | **_** | **c** | **D** | **c** | **D** | **a** | **_** | **c** | **_** | **a** | **D** | **c** | **D** | **a** | **.** | **.** | **.** | **.** | **.** | **.** | | **I V E I E E V P S Q T N N** | | |
|  | **AF191065** | **N N E F Q E I N E D** | **1** | **D** | **b** | **_** | **c** | **D** | **c** | **D** | **a** | **_** | **c** | **D** | **c** | **D** | **a** | **.** | **.** | **.** | **.** | **.** | **.** | **.** | **.** | **.** | **.** | | **I V E I E E V P S Q T N N** | | |
|  | **AF191066** | **N N E F Q E I N E D** | **1** | **D** | **b** | **_** | **a** | **_** | **c** | **_** | **a** | **_** | **c** | **E** | **a** | **.** | **.** | **.** | **.** | **.** | **.** | **.** | **.** | **.** | **.** | **.** | **.** | | **I V E I E E V P S Q T N N** | | |
|  | **AF191067** | **N N E F Q E I N E D** | **1** | **D** | **b** | **_** | **c** | **_** | **c** | **_** | **a** | **_** | **c** | **_** | **a** | **_** | **a** | **_** | **c** | **E** | **a** | **D** | **a** | **.** | **.** | **.** | **.** | | **I V E I E E V P S Q** | | |
|  | **AY138510** | **N N E F Q E I N E D** | **1** | **D** | **_** | **_** | **_** | **_** | **c** | **_** | **a** | **_** | **c** | **_** | **a** | **_** | **a** | **_** | **c** | **_** | **c** | **E** | **a** | **.** | **.** | **.** | **.** | | **I V E I E E V T I T K Q I T I E N** | | |
|  | **AY138511** | **N N E F Q E I N E D** | **1** | **D** | **_** | **_** | **_** | **_** | **c** | **_** | **a** | **_** | **c** | **_** | **a** | **_** | **a** | **_** | **c** | **_** | **c** | **E** | **a** | **.** | **.** | **.** | **.** | | **I V E I E E V T I T K Q I T I E N** | | |
|  | **M59706** | **N N E F Q E I N E D** | **1** | **D** | **b** | **_** | **a** | **D** | **b** | **_** | **a** | **_** | **b** | **_** | **a** | **_** | **c** | **_** | **a** | **E** | **a** | **_** | **c** | **_** | **c** | **.** | **.** | | **I V E I E E V P S Q T N N** | | |
| **Group-B** | **2011A** | **N N E F Q E I N E D** | **1** | **D** | **b** | **_** | **a** | **_** | **c** | **_** | **a** | **_** | **a** | **_** | **c** | **E** | **a** | **_** | **c** | **.** | **.** | **.** | **.** | **.** | **.** | **.** | **.** | | **I V E I E E V P S Q T N N** | | |
|  | **2011B** | **N N E F Q E I N E D** | **1** | **D** | **b** | **_** | **a** | **_** | **c** | **_** | **a** | **_** | **c** | **_** | **a** | **_** | **c** | **.** | **.** | **.** | **.** | **.** | **.** | **.** | **.** | **.** | **.** | | **I V E I E E V P S Q T N N** | | |
|  | **2011C** | **N N E F Q E I N E D** | **1** | **D** | **b** | **_** | **a** | **_** | **c** | **_** | **a** | **_** | **c** | **_** | **a** | **_** | **c** | **.** | **.** | **.** | **.** | **.** | **.** | **.** | **.** | **.** | **.** | | **I V E I E E V P S Q T N N** | | |
|  | **2011D** | **N N E F Q E I N E D** | **1** | **D** | **b** | **_** | **a** | **_** | **c** | **_** | **a** | **_** | **c** | **_** | **a** | **_** | **c** | **.** | **.** | **.** | **.** | **.** | **.** | **.** | **.** | **.** | **.** | | **I V E I E E V P S Q T N N** | | |
|  | **2011F** | **N N E F Q E I N E D** | **1** | **D** | **b** | **_** | **a** | **_** | **c** | **_** | **a** | **_** | **a** |  | **c** | **_** | **a** | **_** | **a** | **_** | **c** | **E** | **a** | **_** | **c** | **.** | **.** | | **I V E I E E V P S Q T N N** | | |
|  | **2011G** | **N N E F Q E I N E D** | **1** | **D** | **b** | **_** | **a** | **_** | **d** | **_** | **a** | **D** | **a** |  | **a** | **D** | **a** | **.** | **.** | **.** | **.** | **.** | **.** | **.** | **.** | **.** | **.** | | **I V E I E E V P S Q T N N** | | |
|  | **2011H** | **N N E F Q E I N E D** | **1** | **D** | **b** | **_** | **c** | **D** | **c** | **D** | **a** | **_** | **c** | **D** | **c** | **D** | **a** | **_** | **c** | **_** | **a** | **D** | **a** |  |  | **.** | **.** | | **I V E I E E V P S Q T N N** | | |
|  | **2011I** | **N N E F Q E I N E D** | **1** | **D** | **b** | **_** | **c** | **D** | **c** | **D** | **a** | **_** | **a** |  | **c** | **E** | **a** | **.** | **.** | **.** | **.** | **.** | **.** | **.** | **.** | **.** | **.** | | **I V E I E E V P S Q T N N** | | |
|  | **2011J** | **N N E F Q E I N E D** | **1** | **D** | **b** | **_** | **a** | **_** | **c** | **_** | **a** | **_** | **c** | **_** | **a** | **_** | **c** | **.** | **.** | **.** | **.** | **.** | **.** | **.** | **.** | **.** | **.** | | **I V E I E E V P S Q T N N** | | |
|  | **2011K** | **N N E F Q E I N E D** | **1** | **D** | **b** | **_** | **a** | **_** | **c** | **_** | **a** | **_** | **c** | **_** | **a** | **_** | **c** | **E** | **a** | **.** | **.** | **.** | **.** | **.** | **.** | **.** | **.** | | **I V** | | |
|  | **2011N** | **N N K F Q E I N E D** | **1** | **D** | **b** | **_** | **a** | **_** | **d** | **_** | **a** | **D** | **a** | **_** | **a** | **D** | **a** | **.** | **.** | **.** | **.** | **.** | **.** | **.** | **.** | **.** | **.** | | **I V E I E E V P S Q T N N** | | |
|  | **2011O** | **N N K F Q E I N E D** | **1** | **D** | **b** | **_** | **a** | **_** | **d** | **_** | **a** | **D** | **a** | **_** | **a** | **D** | **a** | **.** | **.** | **.** | **.** | **.** | **.** | **.** | **.** | **.** | **.** | | **I V E I E E V P S Q T N N** | | |
|  | **2011P** | **N N K F Q E I N E D** | **1** | **D** | **b** | **_** | **a** | **_** | **d** | **_** | **a** | **D** | **a** | **_** | **a** | **D** | **a** | **.** | **.** | **.** | **.** | **.** | **.** | **.** | **.** | **.** | **.** | | **I V E I E E V P S Q T N N** | | |
|  | **2011Q** | **N N E F Q E I N E D** | **1** | **D** | **b** | **_** | **a** | **_** | **a** | **_** | **a** | **_** | **c** | **E** | **a** | **.** | **.** | **.** | **.** | **.** | **.** | **.** | **.** | **.** | **.** | **.** | **.** | | **I V E I E E V P S Q T N N** | | |
| **Group-A** | **2005A** | **N N E F Q E I N E D** | **1** | **D** | **b** | **_** | **a** | **_** | **c** | **_** | **a** | **_** | **a** | **_** | **c** | **_** | **a** | **.** | **.** | **.** | **.** | **.** | **.** | **.** | **.** | **.** | **.** | | **I V E I E E V P S Q T N N** | | |
|  | **2005B** | **N N E F Q E I N E D** | **1** | **D** | **b** | **_** | **c** | **_** | **a** |  | **c** | **_** | **a** | **_** | **c** | **_** | **a** |  | **c** | **E** | **a** | **.** | **.** | **.** | **.** | **.** | **.** | | **I V E I E E V P S Q T N N** | | |
|  | **2005C** | **N N E F Q E I N E D** | **1** | **D** | **b** | **_** | **a** | **_** | **c** | **_** | **a** | **_** | **c** | **_** | **a** | **E** | **c** | **E** | **a** | **.** | **.** | **.** | **.** | **.** | **.** | **.** | **.** | | **I V E I E E V P S Q T N N** | | |
|  | **2005D** | **N N E F Q E I N E D** | **1** | **D** | **b** | **D** | **c** | **_** | **c** | **_** | **a** | **_** | **a** | **_** | **c** | **_** | **c** | **_** | **a** | **_** | **a** | **D** | **a** | **.** | **.** | **.** | **.** | | **I V E I E E V P S Q T N N** | | |
|  | **2005E** | **N N E F Q E I N E D** | **1** | **D** | **b** | **_** | **a** | **_** | **c** | **_** | **a** | **_** | **c** | **_** | **a** | **E** | **c** | **E** | **a** | **.** | **.** | **.** | **.** | **.** | **.** | **.** | **.** | | **I V E I E E V P S Q T N N** | | |
|  | **2005G** | **N N E F Q E I N E D** | **1** | **D** | **b** | **_** | **a** | **_** | **c** | **_** | **a** | **_** | **c** | **_** | **a** | **_** | **c** | **E** | **a** | **.** | **.** | **.** | **.** | **.** | **.** | **.** | **.** | | **I V E I E E** | | |
|  | **2005O** | **I N E D** | **1** | **D** | **b** | **_** | **a** | **_** | **c** | **_** | **a** | **_** | **c** | **_** | **a** | **_** | **c** | **E** | **a** | **.** | **.** | **.** | **.** | **.** | **.** | **.** | **.** | | **I V E I E E V P S Q T N N** | | |
|  | **2005P** | **N N E F Q E I N E D** | **1** | **D** | **b** | **_** | **a** | **_** | **c** | **_** | **a** | **_** | **c** | **_** | **a** | **_** | **c** | **E** | **a** | **.** | **.** | **.** | **.** | **.** | **.** | **.** | **.** | | **I V E I E E V P S Q T N N** | | |
|  | **2005F** | **N N E F Q E I N E D** | **1** | **D** | **b** | **_** | **a** | **_** | **c** | **_** | **a** | **_** | **c** | **_** | **a** | **_** | **c** | **_** | **a** | **_** | **c** | **_** | **a** | **_** | **c** | **E** | **a** | | **I V E I E E V P S Q T N N** | | |
|  |  |  |  |  |  |  |  |  |  |  |  |  |  |  |  |  |  |  |  |  |  |  |  |  |  |  |  | |  |  | |
|  | | |  |  |  |  |  |  |  |  |  |  |  |  |  |  |  |  |  |  |  |  |  |  |  |  |  | |  |  | |
|  |  |  | **D** | **K** | **S** | **A** | **H** | **I** | **Q** | **H** | **E** | **I** | **V** | **E** | **V** | **E** | **E** | **I** | **L** | **P** | **E** |  | **1** |  |  | | | | | |  |
|  |  |  | **D** | **K** | **N** | **E** | **K** | **G** | **Q** | **H** | **E** | **I** | **V** | **E** | **V** | **E** | **E** | **I** | **L** | **P** | **E** |  | **a** |  |  |  |  |  |  |  |  |
|  |  |  | **D** | **K** | **N** | **E** | **K** | **V** | **E** | **H** | **E** | **I** | **V** | **E** | **V** | **E** | **E** | **I** | **L** | **P** | **E** |  | **b** |  |  |  |  |  |  |  |  |
|  |  |  | **D** | **K** | **N** | **E** | **K** | **V** | **Q** | **H** | **E** | **I** | **V** | **E** | **V** | **E** | **E** | **I** | **L** | **P** | **E** |  | **c** |  |  |  |  |  |  |  |  |
|  |  |  | **D** | **K** | **N** | **E** | **K** | **V** | **E** | **H** | **E** | **I** | **V** | **E** | **V** | **D** | **E** | **I** | **L** | **P** | **E** |  | **d** |  |  |  |  |  |  |  |  |
|  |  |  | **Types of repeat sequence (19 Amino acids pairs) in R 2 region** | | | | | | | | | | | | | | | | | | | **Unit code** | | |  |  |  |  |  |  |  |

**Additional file 1 Distribution of amino acid repeat unit (AAU) of GLURP R2 region of field isolates and NCBI sequences of *Plasmodium falciparum.***
