# Supplementary material for: Genetic polymorphism and amino acid sequence variation in Plasmodium falciparum GLURP R2 repeat region in Assam, India, at an interval of five years
Source: Malar J. 2014 Nov 21;13:450. doi: 10.1186/1475-2875-13-450 (PMC4256832; doi:10.1186/1475-2875-13-450)
Supplement: Supplementary file 2 — Additional file 2:Arrangement of amino acid repeat order (AAUO) and haplotype diversity of GLURP R 2 region of P. falciparum in field isolates and NCBI sequence database.(DOCX 39 KB) [file 12936_2014_3613_MOESM2_ESM.docx]

|  | **Types of amino acid sequence unit order** | | | | | | | | | | | **Frequency** | | | | | **Amino acid repeat sequence unit (AAU)** | | | | | **Amino acid repeat sequence unit order (AAUO)** | | | | | | | **Extra D and E amino acid** | | | |  |
| --- | --- | --- | --- | --- | --- | --- | --- | --- | --- | --- | --- | --- | --- | --- | --- | --- | --- | --- | --- | --- | --- | --- | --- | --- | --- | --- | --- | --- | --- | --- | --- | --- | --- |
|  | **TYPES** |  |  |  | **Order of Repeat Unit** | | | | | | | | | **n** | | | **a** | **b** | **c** | **d** | **TOTAL** | **T** | **U** | **V** | **W** | **X** | **Y** | **Z** | **TOTAL** | **D** | **E** | **TOTAL** | |
|  | **Order of amino acid sequence in R2 repeat region of *P. falciparum* GLURP in Group-A (collected in 2005; N=9)** | | | | | | | | | | | | | | | | | | | | | | | | | | | | | | | | |
| **Group-A** | T8 | I N E D | 1 | D | X | Y | Y | Yx | . | . | . | . | IV | G,O,P | | | 4 | 1 | 3 | 0 | 8 | 0 | 0 | 0 | 0 | 1 | 3 | 0 | 4 | 0 | 1 | 1 | |
|  | T9 | I N E D | 1 | D | X | Y | Y | E | Yx | . | . | . | IV | C,E | | | 4 | 1 | 3 | 0 | 8 | 0 | 0 | 0 | 0 | 1 | 3 | 0 | 4 | 0 | 2 | 2 | |
|  | T10 | I N E D | 1 | D | X | Y | Z | a | . | . | . | . | IV | A | | | 4 | 1 | 2 | 0 | 7 | 0 | 0 | 0 | 0 | 1 | 1 | 1 | 3 | 0 | 0 | 0 | |
|  | T11 | I N E D | 1 | D | X | Y | Y | Y | Y | Yx | . | . | IV | F | | | 6 | 1 | 5 | 0 | 12 | 0 | 0 | 0 | 0 | 1 | 5 | 0 | 6 | 0 | 1 | 1 | |
|  | T12 | I N E D | 1 | D | Wy | Y | Z | Y | **Vy** | . | . | . | IV | D | | | 5 | 1 | 4 | 0 | 10 | 0 | 0 | 1 | 1 | 0 | 2 | 1 | 5 | 2 | 0 | 2 | |
|  | T13 | I N E D | 1 | D | W | Z | Z | Z | E | a | . | . | IV | B | | | 4 | 1 | 4 | 0 | 9 | 0 | 0 | 0 | 1 | 0 | 0 | 3 | 4 | 0 | 1 | 1 | |
|  |  | | | | | | | | | | | | **Total** | | | | **39** | **9** | **30** | **0** | **78** | **0** | **0** | **1** | **2** | **7** | **23** | **5** | **38** | **2** | **9** | **11** | |
|  |  |  |  |  |  |  |  |  |  |  |  |  | **Mean** | | | | **4.3** | **1** | **3.3** | **0** | **8.7** | **0** | **0** | **0.1** | **0.2** | **0.8** | **2.6** | **0.6** | **4.2** | **0.2** | **1** | **1.2** | |
|  |  |  |  |  |  |  |  |  |  |  |  |  | **St. deviation (±)** | | | | **0.7** | **0** | **0.9** | **0** | **1.5** | **0** | **0** | **0.3** | **0.4** | **0.4** | **1.4** | **1** | **0.8** | **0.7** | **0.7** | **0.7** | |
|  | **Order of amino acid sequence in R 2 repeat region of *P. falciparum* GLURP in Group-B (collected in 2011; N=14)** | | | | | | | | | | | | | | | | | | | | | | | | | | | | | | | | |
| **Group-B** | T14 | I N E D | 1 | D | X | Y | Y | c | . | . | . | . | IV | B,C,D,J | | | 3 | 1 | 3 | 0 | 7 | 0 | 0 | 0 | 0 | 1 | 2 | 0 | 3 | 0 | 0 | 0 | |
|  | T8 | I N E D | 1 | D | X | Y | Y | Yx | . | . | . | . | IV | K | | | 4 | 1 | 3 | 0 | 8 | 0 | 0 | 0 | 0 | 1 | 3 | 0 | 4 | 0 | 1 | 1 | |
|  | T15 | I N E D | 1 | D | X | Y | Z | E | Z | . | . | . | IV | A | | | 4 | 1 | 3 | 0 | 8 | 0 | 0 | 0 | 0 | 1 | 1 | 2 | 4 | 0 | 1 | 1 | |
|  | T16 | I N E D | 1 | D | X | **T** | D | V | D | a | . | . | IV | G,N,O,P | | | 5 | 1 | 0 | 1 | 7 | 1 | 0 | 1 | 0 | 1 | 0 | 0 | 3 | 2 | 0 | 2 | |
|  | T17 | I N E D | 1 | D | X | V | Yx | . | . | . | . | . | IV | Q | | | 4 | 1 | 1 | 0 | 6 | 0 | 0 | 1 | 0 | 1 | 1 | 0 | 3 | 0 | 1 | 1 | |
|  | T18 | I N E D | 1 | D | W | D | Yz | Uy | D | Z | Vy | . | IV | H | | | 4 | 1 | 5 | 0 | 10 | 0 | 1 | 1 | 1 | 0 | 1 | 1 | 5 | 5 | 0 | 5 | |
|  | T19 | I N E D | 1 | D | W | D | Yz | Z | E | a | . | . | IV | I | | | 3 | 1 | 3 | 0 | 7 | 0 | 0 | 0 | 1 | 0 | 1 | 1 | 3 | 2 | 1 | 3 | |
|  | T20 | I N E D | 1 | D | X | Y | Z | V | Yx | c | . | . | IV | F | | | 6 | 1 | 4 | 0 | 11 | 0 | 0 | 1 | 0 | 1 | 2 | 1 | 5 | 0 | 1 | 1 | |
|  |  | | | | | | | | | | | | **Total** | | | | **57** | **14** | **31** | **4** | **106** | **4** | **1** | **7** | **2** | **12** | **17** | **5** | **48** | **15** | **5** | **20** | |
|  |  |  |  |  |  |  |  |  |  |  |  |  | **Mean** | | | | **4.1** | **1** | **2.2** | **0.3** | **7.6** | **0.3** | **0.1** | **0.5** | **0.1** | **0.9** | **1.2** | **0.4** | **3.4** | **1.1** | **0.4** | **1.4** | |
|  |  |  |  |  |  |  |  |  |  |  |  |  | **St. deviation (±)** | | | | **1** | **0** | **1.7** | **0.5** | **1.3** | **0.5** | **0.3** | **0.5** | **0.4** | **0.4** | **1** | **0.6** | **0.8** | **1.5** | **0.5** | **1.4** | |
|  |  |  |  |  |  |  |  |  |  |  |  |  | **p value** | | | | **0.6** | **nc** | **0.1** | **nc** | **0.1** | **nc** | **nc** | **0.04** | **0.6** | **0.6** | **0.01** | **0.6** | **0.03** | **0.1** | **0.03** | **0.7** | |
|  | **Order of amino acid sequence in R 2 repeat region of *P. falciparum* GLURP from NCBI sequences (N=8)** | | | | | | | | | | | | | | | | | | | | | | | | | | | | | | | | |
| **NCBI sequences** | T1 | I N E D | 1 | D | W | X | Y | X | D | a | . | . | IV | NCBI 1 | | | 4 | 3 | 2 | 0 | 9 | 0 | 0 | 0 | 1 | 2 | 1 | 0 | 4 | 1 | 0 | 1 | |
|  | T2 | I N E D | 1 | D | W | Uy | D | Z | Zy | D | a | . | IV | NCBI 2 | | | 3 | 1 | 5 | 0 | 9 | 0 | 1 | 0 | 1 | 0 | 0 | 2 | 4 | 4 | 0 | 4 | |
|  | T3 | I N E D | 1 | D | W | D | Yz | Uy | D | a | . | . | IV | NCBI 3 | | | 2 | 1 | 4 | 0 | 7 | 0 | 1 | 0 | 1 | 0 | 1 | 0 | 3 | 4 | 0 | 4 | |
|  | T4 | I N E D | 1 | D | X | Y | Yx | . | . | . | . | . | IV | NCBI 4 | | | 3 | 1 | 2 | 0 | 6 | 0 | 0 | 0 | 0 | 1 | 2 | 0 | 3 | 0 | 1 | 1 | |
|  | T5 | I N E D | 1 | D | W | Y | Y | Z | E | Vy | . | . | IV | NCBI 5 | | | 5 | 1 | 4 | 0 | 10 | 0 | 0 | 1 | 1 | 0 | 2 | 1 | 5 | 1 | 1 | 2 | |
|  | T6 | I N E D | 1 | D | _ | Y | Y | Z | Yx | . | . | . | IV | NCBI 6-7 | | | 4 | 0 | 4 | 0 | 8 | 0 | 0 | 0 | 0 | 0 | 3 | 1 | 4 | 0 | 1 | 1 | |
|  | T7 | I N E D | 1 | D | X | D | X | X | Y | E | Z | c | IV | NCBI 8 | | | 5 | 3 | 3 | 0 | 11 | 0 | 0 | 0 | 0 | 3 | 1 | 1 | 5 | 1 | 1 | 2 | |
|  |  |  |  |  |  |  |  |  |  |  |  |  | **Total** | | | | **30** | **10** | **28** | **0** | **68** | **0** | **2** | **1** | **4** | **6** | **13** | **6** | **32** | **11** | **5** | **16** | |
|  |  | **c _ c = U** | | |  |  |  |  |  |  |  |  | **Mean** | | | | **3.8** | **1.3** | **3.5** | **0** | **8.5** | **0** | **0.3** | **0.1** | **0.5** | **0.8** | **1.6** | **0.8** | **4** | **1.4** | **0.6** | **2** | |
|  |  | **a _ a = V** | | |  |  | **c D c = Uy** | | | |  |  | **St. deviation (±)** | | | | **1** | **1.2** | **1.1** | **0** | **1.6** | **0** | **0.5** | **0.4** | **0.5** | **1.2** | **1.1** | **0.7** | **0.8** | **1.7** | **0.5** | **1.3** | |
|  |  | **b _ c = W** | | |  |  | **a D a = Vy** | | | |  |  | **Order of amino acid sequence in R 2 repeat region of *P. falciparum* GLURP collected in 2005 (N=9) +2011 (N=14) [Total=23]** | | | | | | | | | | | | | | | | | | | | |
|  |  | **b _ a = X** | | |  |  | **b D c = Wy** | | | |  |  | **Total** | | | | **96** | **23** | **61** | **4** | **184** | **4** | **1** | **8** | **4** | **19** | **40** | **10** | **86** | **17** | **14** | **31** | |
|  |  | **c _ a = Y** | | |  |  | **c D a = Yz** | | | |  |  | **Mean** | | | | **4.2** | **1** | **2.7** | **0.2** | **8** | **0.2** | **0.04** | **0.3** | **0.2** | **0.8** | **1.7** | **0.4** | **3.7** | **0.7** | **0.6** | **1.3** | |
|  |  | **a _ c = Z** | | |  |  | **c E a = Yx** | | | |  |  | **St. deviation (±)** | | | | **0.9** | **0** | **1.5** | **0.4** | **1.5** | **0.4** | **0.2** | **0.5** | **0.4** | **0.4** | **1.3** | **0.8** | **0.9** | **1.3** | **0.7** | **1.1** | |
|  |  | **d _ a = T** | | |  |  | **a D c = Zy** | | | |  |  | **p value** | | | | **0.3** | **nc** | **0.2** | **nc** | **0.4** | **nc** | **0.04** | **0.3** | **0.1** | **>0.9** | **0.8** | **0.2** | **0.4** | **0.2** | **>0.9** | **0.2** | |
|  |  |  |  |  |  |  |  |  |  |  |  |  |  | |  |  |  |  |  |  |  |  |  |  |  |  |  |  |  |  |  |  |  |
|  | **nc= not calculatable** | | | | | | | | | | | | | | | | | | | | | | | | | | | | | | | | |

**Additional file 2 Arrangement of amino acid repeat order (AAUO) and haplotype diversity of GLURP R 2 region of *P. falciparum* in field isolates and NCBI sequence database.**
